# Investigating the Antibody Imprinting Hypothesis among Canadian Paramedics after SARS-CoV-2 Omicron Variant Circulation

**DOI:** 10.4049/immunohorizons.2400010

**Published:** 2024-02-22

**Authors:** Michael Asamoah-Boaheng, Brian Grunau, Mohammad Ehsanul Karim, Iryna Kayda, Justin Yap, Katherine Bessai, David M. Goldfarb

**Affiliations:** * Department of Emergency Medicine, University of British Columbia, Vancouver, British Columbia, Canada; † Centre for Advancing Health Outcomes, University of British Columbia, Vancouver, British Columbia, Canada; ‡ British Columbia Resuscitation Research Collaborative, Vancouver, British Columbia, Canada; § School of Population and Public Health, University of British Columbia, Vancouver, British Columbia, Canada; ¶ Experimental Medicine Graduate Program, Faculty of Medicine, University of British Columbia, Vancouver, British Columbia, Canada; ǁ Faculty of Science, University of British Columbia, Vancouver, British Columbia, Canada; # British Columbia Emergency Health Services, British Columbia, Canada; ** Department of Pathology and Laboratory Medicine, University of British Columbia, Vancouver, British Columbia, Canada

## Abstract

Recent research has highlighted the Omicron variant’s capacity to evade immune protection conferred by wild-type (WT) mRNA vaccines. Despite this observation, the potential involvement of antigenic sin phenomena remains unclear. Our hypothesis posited that a greater number of prior WT vaccine doses might lead to reduced anti-Omicron neutralization Abs following Omicron infection. To investigate this, we analyzed blood samples from human participants in the COVID-19 Occupational Risk, Seroprevalence, and Immunity among Paramedics (CORSIP) study who had received at least one WT mRNA vaccine before contracting Omicron. The exposure variable was the number of WT mRNA vaccines administered, and the outcome was the angiotensin-converting enzyme 2 (ACE-2) percent inhibition specific to the BA.4/BA.5 Omicron Ag. Contrary to expectations, our findings revealed that more WT-based vaccines were associated with an enhanced Omicron-specific immune response.

## Introduction

The emergence of the highly immune evasive SARS-CoV-2 Omicron variant has had significant impacts on the course of the pandemic. Given that at the time of its emergence, much of the global population had received multiple vaccinations with platforms targeting the original Wuhan Hu-1 SARS-CoV-2 spike protein (1), questions have arisen as to whether repeated exposures to an ancestral variant Ag through vaccination result in a phenomenon termed immunological imprinting (otherwise known as “original antigenic sin”) (2). Immunological imprinting occurs when a host’s immune system has been primed with one Ag (e.g., either through vaccination or infection) but then encounters a modified version of that Ag (e.g., a more contemporary strain of pathogen) but the immune response is “trapped” to some degree in a response that is directed against the original Ag. As a result, the Abs produced by the immune system may not be as effective in neutralizing the new variant, and the individual may be at increased risk of reinfection (3). This has been previously described as a concern with vaccination or infection due to influenza (4) and other RNA viruses (5). In the context of the COVID-19 pandemic, although the original Wuhan strain–based vaccine platform (wild-type [WT]) vaccines have been shown to offer protection against severe disease and hospitalization (6), emerging studies have documented the ability of the Omicron variant to escape the immune protection provided by these vaccines (7). However, whether the antigenic sin phenomena play a role in this is unclear.

We sought to explore whether antigenic sin may be playing a role in humoral immunity to the Omicron strain. We examined immunogenicity against the Omicron variants in individuals who had received varying prior doses of mRNA WT vaccines and immunity from previous Omicron infection. We hypothesized that more preceding WT vaccine doses may result in lower anti-Omicron neutralization Abs subsequent to Omicron infection.

## Materials and Methods

### Study design and data source

This study employed blood samples from the COVID-19 Occupational Risks, Seroprevalence, and Immunity among Paramedics in Canada (CORSIP) observational cohort study. CORSIP is a longitudinal prospective study examining the workplace risks and seroprevalence of SARS-CoV-2 exposure among paramedics working in the Canadian provinces of British Columbia, Alberta, Ontario, Saskatchewan, and Manitoba. At 6-mo intervals, participants provided blood samples (tested with nucleocapsid and spike assays) and questionnaire data on vaccination status, demographic characteristics, medical history, type of vaccine administered, and other lifestyle factors. The University of British Columbia (H20-03620) and University of Toronto (40435) Research Ethics Boards provided ethics approval for this study. Participants also provided electronic consent upon enrolment.

### Study participants

For this investigation, we included blood samples from CORSIP participants who 1) received at least one WT mRNA vaccine before Omicron infection, and 2) were infected with Omicron SARS-CoV-2 after vaccination but before blood sample collection. Omicron infection was defined as a positive nucleic acid amplification viral test or a positive rapid Ag test after December 26, 2021. We assumed that all SARS-CoV-2 infections after December 26, 2021, were Omicron strain infections, given that at this time point, >95% of Canadian SARS-Cov-2 infections were due to the Omicron strain (8, 9). We excluded individuals 1) who had pre-Omicron SARS-Cov-2 infections (on or before December 25, 2021), defined as either a positive nucleic acid amplification test, a positive rapid Ag test, or previous reactive serology using the Elecsys nucleocapsid anti–SARS-CoV2 assay (Roche Diagnostics, Indianapolis, IN; 2) who received any vaccines other than monovalent WT mRNA vaccines; and 3) cases who had an Omicron infection within 30 d of the blood collection.

### Exposure variable

The exposure variable was the number of WT mRNA vaccines received.

### Outcome variable

The primary outcome was angiotensin-converting enzyme 2 (ACE-2) percent inhibition to the BA.4/BA.5 Omicron Ag.

### Serological testing

All blood samples were tested with 1) the V-PLEX SARS-CoV-2 panel 28 ACE-2 kit (Meso Scale Discovery, Rockville, MD) to measure percent inhibition of ACE-2 binding to Omicron (B.1.617.2) BA.4/BA.5 sublineages, and 2) the Elecsys anti–SARS-CoV-2 nucleocapsid assay (Roche Diagnostics, Indianapolis, IN) to identify anti-nucleocapsid protein Abs for detecting proceeding SARS-CoV-2 infection.

### Statistical analysis

We described continuous variables with mean and SD, or median with interquartile range (IQR), and summarized categorical variables with counts and percentages. We used the multiple linear regression model to investigate the association between the number of WT mRNA vaccines received (discrete continuous variable) and the log-transformed immune response measured by ACE-2 inhibitions to BA.4/5 Ag. First, we performed an unadjusted simple linear regression (included only the exposure and outcome variables in the model). Previous studies have identified age, sex, smoking status/tobacco use, comorbidities, and race as confounding factors for investigating the association between COVID-19 vaccination and immune response (10–13). Other investigations have identified age, comorbidities, and timing of infection-to-blood collection as risk factors for immune response (outcome) (10, 11, 14). Based on these data, we used the following adjustment covariates in the model: female sex (at birth), race (racialized [including Chinese, Blacks, Filipinos, Latin Americans, Southeast Asians] versus Whites), tobacco use, E-cigarette use, influenza vaccination, Omicron infection-to-blood collection interval (days), and participants’ medical history (including asthma, diabetes, hypertension, and cancer [each used as individual binary variables]). Statistical significance was classified at a significance level of *p *<* *0.05. In a sensitivity analysis, we categorized the number of vaccines received as one to two vaccines versus more than two vaccines. The number of vaccines was dichotomized to compare those who received only the primary series of two vaccines, to those who received additional booster doses (15, 16).

## Results

The study included a total of 257 paramedics who received at least one WT mRNA vaccine, then experienced an Omicron infection, and then provided a blood sample. The mean participant age was 39 y. Participants characteristics, stratified by the number of WT vaccines received, are shown in Table I. Overall, 166 (65%) received three vaccines before the Omicron infection, and 91 (35%) received one or two vaccines. The median interval between the Omicron infection and blood collection was 106 d (IQR 66, 169). The median ACE-2 percent inhibition to BA.4/5 Omicron Ag was 91% (IQR 80, 97).

**Table I.** Study characteristics and outcomes

		No. of mRNA Monovalent Vaccine Doses	
Variables	Full Cohort (*n* = 257)	Vaccines (*n* = 91) (35)	More than Two Vaccines (*n* = 166) (65)
Age (y), mean (SD)	39 (10)	38 (10)	40 (10)
Female sex (at birth)	107 (42)	36 (40)	71 (43)
Race			
Racialized*^a^*	11 (4.3)	6.0 (7.0)	5.0 (3.0)
White	246 (96)	85 (93)	161 (97)
Tobacco use, *n* (%)	13 (5.1)	6.0 (7.0)	7.0 (4.2)
E-cigarette, *n* (%)	15 (6)	7.0 (8.0)	8.0 (5.0)
Influenza vaccination*, n* (%)	175 (68)	44 (48)	131 (79)
Medical history			
Hypertension	21 (8)	6 (7)	15 (9)
Diabetes	5 (2)	0 (0)	5 (3)
Asthma	25 (10)	8 (9)	17 (10)
Heart diseases	3 (1)	1 (1)	2 (1)
Kidney diseases	1 (0)	1 (1)	0 (0)
Liver diseases	2 (1)	0 (0)	2 (1)
Cancer	5 (2)	2 (2)	3 (2)
Vaccine type, *n* (%)			
Dose 1 (BNT162b2)	193 (75)	68 (75)	125 (75)
Dose 1 (mRNA-1273)	63 (24)	22 (24)	41 (25)
Dose 1 (missing)	1.0 (1.0)	1 (1)	0 (0)
Dose 2 (BNT162b2)	187 (73)	65 (71)	122 (74)
Dose 2 (mRNA-1273)	67 (26)	23 (25)	44 (27)
Dose 2 (missing)	3.0 (1.0)	2 (2)	1 (1)
Dose 3 (BNT162b2)	101 (39)	5 (6)	96 (58)
Dose 3 (mRNA-1273)	73 (28)	3 (3)	70 (42)
Dose 3 (missing)	83 (32)	83 (91)	0 (0)
Vaccine dosing interval			
V1-to-V2, median (IQR)	45 (40, 99)	61 (40, 104)	—
V2-to-V3, median (IQR)	247 (212, 269)	—	247 (212, 269)
V1-to-BC, median (IQR)	545 (520, 563)	539 (494, 549)	—
V2-to-BC, median (IQR)	480 (419, 518)	549 (531, 568)	—
V3-to-BC, median (IQR)	242 (211, 268)	—	242 (211, 268)
Median (IQR) date of Omicron infection	April 13, 2022 (February 2, 2022, May 20, 2022)	March 1, 2022 (January 15, 2022, April 26, 2022)	March 4, 2022 (February 28, 2022, May 30, 2022)
Omicron infection-to-BC, median (IQR)	106 (66, 169)	139 (78,190)	94 (61, 151)
Date of BC, Median (IQR)	August 4, 2022 (July 15, 2022, August 23, 2022)	August 13, 2022 (July 15, 2022, August 17, 2022)	August 2, 2022 (July 16, 2022, August 26, 2022)
ACE-2 inhibition results			
ACE-2 percent inhibition to BA.4/5 Ag, median (IQR)	91 (80, 97)	86 (71, 95)	95 (83, 98)

a Racialized group includes Chinese, Blacks, Filipinos, Latin Americans, and Southeast Asians.

Table II shows the results of the unadjusted and adjusted multivariate regression analysis, demonstrating that the number of WT mRNA vaccines received was associated with increasing levels of ACE-2 inhibition to BA.4/5 Ag. We found similar results when examining one to two versus more than two vaccines (Table II).

**Table II.** Multivariate analysis of association between number of vaccine doses and ACE-2 inhibition to RBD BA4.BA5

Model	Variables	Unadjusted Coefficient β (95% CI)	Adjusted Coefficient β (95% CI)
Model 1	Main Exposure variable		
	No. of vaccine doses	0.048 (0.019, 0.077)**	0.047 (0.010, 0.083)**
Model 2	No. of doses (categorial)		
	One to two vaccines	Reference	Reference
	More than two vaccines	0.057 (0.024, 0.090)**	0.052 (0.016, 0.087)**

Models 1 and 2 were adjusted for confounders and risk factors, including female sex, age, tobacco use, e-cigarette, influenza vaccination, race, Omicron infection-to-blood collection, medical history (including hypertension, diabetes, asthma, and cancer). **p < 0.05.

## Discussion

We examined individuals who had received only mRNA WT-directed monovalent vaccines, then experienced an Omicron infection, and then provided a blood sample. We investigated the antigenic sin hypothesis, which hypothesizes that more WT-based vaccines prior to an Omicron infection would lead to a relatively attenuated Omicron-specific humoral immune response postinfection (17). Our findings, however, revealed that more WT-based vaccines were associated with an enhanced Omicron-specific immune response, as measured by the ACE-2 inhibition to BA.4/5 Omicron Ag. This suggests that multiple doses of the COVID-19 vaccines plus immunity from Omicron infection may lead to a stronger immune response against reinfection to the Omicron variant. Our findings corroborate previous studies that investigated protective effectiveness of hybrid immunity against the Omicron variant (18). Overall, we did not find evidence of the antigenic sin phenomenon with respect to humoral immunity in our study, and our results were contrary with those of studies supporting the phenomenon (19). Furthermore, the current study suggests that repeated doses of an “antigenically distant” vaccine did not reduce humoral immune responses to Omicron virus infection, but rather enhanced the pseudoneutralization immune response. This not only expands our understanding of immune responses to Omicron but also offers insights into the potential benefits of a multilayered vaccination approach in the face of emerging coronavirus variants. Although this has been studied more extensively in influenza virus immune responses, there is still a relative paucity of evidence for SARS-CoV-2 (20).

The observed immune response can be attributed to the potential boost in neutralizing Abs provided by the additional vaccine doses and a potential additional immune response provided from previous Omicron variant infection. The increased ACE-2 percent inhibition observed in our study indicates a higher degree of neutralization of the Omicron variant, which may help explain the observed reduction in risk of progression to severe disease. The timing of the blood collection in relation to the Omicron infection may have also influenced the observed immune response; that is, the median days from infection to blood collection was 106, indicating that the participants had a substantial period to mount a humoral immune response.

Although our study focused on paramedics, who are more likely to have a higher risk of exposure to SARS-Cov-2, the findings may have implications for the general population as well. Understanding the impact of multiple vaccine doses on immune response can inform vaccination strategies, particularly in the context of a pandemic due to the emergence a novel RNA respiratory virus where it can be expected that there will likely be significant and ongoing genetic and antigenic modification.

Our study has some limitations. First, our study included middle-aged adult Canadian paramedics, whose immune response may differ from other individuals or settings, potentially limiting the generalizability of the results to other populations. We examined the immune response based on ACE-2 percent inhibitions to BA.4/5 Omicron Ag, and further investigations into other markers of immune response, such as T cell responses, could provide additional understanding. Our study may have been influenced by residual or unmeasured confounding. For example, varying exposure levels to SARS-CoV-2, access or adherence to personal protective equipment, and geographical differences based on work location may have introduced variability in exposure risk.

In conclusion, an increasing number of monovalent mRNA COVID-19 vaccine doses prior to Omicron variant infection was associated with higher levels of postinfection Omicron-specific humoral immunogenicity. These findings are not supportive of the antigenic sin phenomenon playing a role in humoral immunity directed against Omicron variants.
